# Hospital Meetings

**Published:** 1902-02-08

**Authors:** 


### HOSPITAL MEETINGS.

### GENERAL LYING-IN HOSPITAL.

Annual Meeting.

Under the chairmanship of Lord Say and Sele the annual reports of the secretary and medical officer were passed by the governors on Wednesday, 29th ult. After the usual preliminaries the secretary read his report, which was not printed, showing that after a visit from Lord Duncannon and Sir Thomas Smith, a donation had been at once received from King Edward's Hospital Fund. The necessary repairs with regard to drainage were being carried out, and they had every reason to believe that the money was being spent on useful schemes. Other improvements were being done, and the hospital had instituted a connection with the telephone. Eighty-one nurses and 41 midwives had been trained. Pupil midwives were now required to have had a previous general training before admission. Thanks were due to Mrs. Johnson for her kindness in receiving both mothers and babies, and also to Mrs. Dakin, who, by a sale of work, realised a large sum for many objects on which the committee did not feel justified in expending the regular funds: it was their aim to act on a business footing, and to discriminate between capital and income. The balance-sheet showed the sum of £200 as extraordinary expenditure; a great deal more than this would have to be spent next year. Dr. Dakin read the medical report. There had been 497 patients during the year; of these 4 had died. General progress had been maintained, and although some of the wards had had to be closed, the number of patients was only 3 less than in 1900. Five hundred and two children were born; of these 47 were born dead, 25 died soon after birth, and 430 left alive and well; 1,887 women were delivered in their own homes. Of the 4 patients who died, 1 was brought into the hospital in an unconscious condition and died from puerperal convulsions 16 hours after delivery without recovering consciousness; the other 3 deaths resulted from intercurrent disease from which the patients were suffering prior to admission: (1) advanced pulmonary phthisis which ended fatally 13 days after labour; (2) cystitis and tubercular kidneys, from which death resulted 5 days later; and (3) acute lobar pneumonia, from which the patient died a fortnight later. The convalescent home at Woking received 29 mothers and 24 infants from the hospital, thereby not only benefiting these patients, but leaving room at the hospital for fresh patients at an earlier date than would otherwise have been possible. Since the opening of the home in 1899, 59 mothers and 45 infants had been admitted. It was resolved to ask Lord Lister, who was unavoidably absent, to re-occupy the position of President; and the meeting ended with the usual votes of thanks.

